# BNP Predicts Chemotherapy-Related Cardiotoxicity and Death: Comparison with Gated Equilibrium Radionuclide Ventriculography

**DOI:** 10.1371/journal.pone.0096736

**Published:** 2014-05-06

**Authors:** Dorthe Skovgaard, Philip Hasbak, Andreas Kjaer

**Affiliations:** 1 Cluster for Molecular Imaging, University of Copenhagen, Copenhagen, Denmark; 2 Department of Clinical Physiology, Nuclear Medicine and PET, Rigshospitalet and University Hospital of Copenhagen, Copenhagen, Denmark; S.G.Battista Hospital, Italy

## Abstract

Cardiotoxicity is a dose-limiting side-effect of cancer chemotherapeutics such as anthracyclines. The drug-induced cardiac toxicity is currently monitored with repeated assessments of the left ventricular ejection fraction (LVEF) using multigated equilibrium radionuclide ventriculography (MUGA) or echocardiography. However, the plasma cardiac biomarker B-type natriuretic peptide (BNP) has been suggested for early identification of cardiac dysfunction. The aim of the study was to compare LVEF obtained by MUGA and plasma BNP as predictors of developing congestive heart failure (CHF) or death in a population of anthracycline-treated cancer patients.

**Methods:**

We prospectively followed 333 cancer patients referred to our department for routine monitoring of LVEF with MUGA and measurement of BNP, January-December 2004. Study end points were hospitalization for CHF and death during follow-up 2004-2010. Data were obtained from the Danish National Patient Registry.

**Results:**

During follow-up (mean 1,360 days), 21 of the patients were admitted to hospital with a diagnosis of CHF and 194 of the patients died. BNP levels were significantly higher and LVEF lower in the group of patients that developed CHF. Using cut-off points of BNP>100 pg/ml (HR 5.5; CI 1.8–17.2; p = 0.003) and LVEF <50% (HR 7.9; CI 3.0–21.4; p<0.001) both significantly predicted CHF. Using the same cut-off points only BNP (HR 1.9; CI 1.3-2.9; p = 0.002) and not LVEF (HR 1.1; CI 0.7–1.8; p = 0.58) was predictive of overall death. In multivariate Cox analysis both BNP and LVEF were independent predictors of CHF while age remained the only independent predictor of overall death.

**Conclusion:**

In cancer patients treated with cardiotoxic chemotherapy both BNP and LVEF can significantly predict subsequent hospitalization with CHF. In addition, BNP and not LVEF has a prognostic value in detecting overall death. This prospective study based on the hitherto largest study population supports BNP as a clinical relevant method for monitoring chemotherapy-related cardiac failure and death.

## Introduction

Cardiotoxicity associated with intensive chemotherapy affects life quality and overall survival of cancer patients. According to estimates cancer survivors in the US and Europe have a higher risk of cardiovascular death than the actual risk of tumor recurrence [1]. The most common way to prevent the immediate chemotherapy-induced cardiotoxicity from leading to irreversible congestive heart failure (CHF) is to monitor cardiac function during and following treatment by estimating left ventricular ejection fraction (LVEF) using radionuclide ventriculography or echocardiography and then if cardiac impairment is detected, treatment dose are lowered or cardioprotectants are added. International oncology guidelines define cardiotoxicity as a decrease in LVEF greater than 10% or an absolute LVEF value below 50% [2]. However, assessment of LVEF is limited by an inability to detect early changes that can predict late declines in cardiac function and therefore there has been a growing interest in identifying circulating biomarkers as reproducible, sensitive and cost effective ways to identify patients in the risk of developing chemotherapy-related cardiomyopathy [2]–[4].

B-type natriuretic peptide (BNP) is one of the suggested biomarkers with well-recognized diagnostic and independent prognostic implications in heart failure patients. BNP is secreted by the ventricles in response to end-diastolic pressure and volume [5]–[7]. Several studies have demonstrated increased plasma BNP levels during cancer therapy with anthracyclines, a group of widely prescribed chemotherpeutic agents with well-known cardiovascular toxicity [6], [7]. However, studies have been conflicting with respect to the correlation between BNP concentration and the standard methods of estimating LVEF [8]–[14]. However, the value of BNP is not to predict LVEF levels but its prognostic and predictive value of developing CHF.

So far, only a few studies have investigated the value of BNP in prediction of clinically endpoints such as manifest cardiac dysfunction following cardiotoxic chemotherapy [6], [7], [13], [15], [16].

Therefore, the aim of the study was to evaluate BNP concentration and LVEF obtained by multigated acquisition equilibrium radionuclide ventriculography (MUGA) as predictors of hospital admission for congestive heart failure (CHF) and mortality (death of any cause) in a population of anthracycline-treated cancer patients.

## Materials and Methods

The study was designed as a prospective study of 333 cancer patients treated with cardiotoxic chemotherapy with the aim of comparing the predictive value of plasma BNP concentration and LVEF assessed by MUGA for detection of cardiotoxicity and death following cancer treatment. We have previously published data from the same cohort comparing baseline BNP and LVEF as determined by MUGA obtained during chemotherapeutic treatment [8]. Now, we present the long-term follow up data. The study population included all patients consecutively referred to the Department of Clinical Physiology, Nuclear Medicine and PET, Rigshospitalet for routine monitoring of LVEF in relation to treatment with cardiotoxic chemotherapy including anthracyclines in the period January-December 2004. All patients underwent MUGA, routinely performed when half of the cumulative dose of anthracyclines has been given, with estimation of LVEF and a blood sample drawn for BNP measurement as described below.

Data concerning hospital admissions of the patients were obtained from the Danish National Patient Register (2004–2010, mean follow up 1,360 days). The Danish National Patient Register (DNPR) is a nationwide register covering all hospital admissions in Denmark. Every Danish citizen has a personal identification number assigned at birth or immigration which is used by public authorities including DNPR. Each hospital admission is routinely reported to DNPR using the international classification of diseases codes for diagnoses and surgical procedure (ICD-10) [17]. Furthermore, data on the causes of death were obtained from the Danish National register of causes of death [18] but in this study only the dates of death and not the specific causes of death were included in analyzes. The study end points were 1) CHF: hospitalization for congestive heart failure (defined as the ICD-10 codes: DI427 and DI50-DI509) and 2) death: all-cause mortality.

### LVEF

LVEF was obtained by multigated acquisition radionuclide ventriculography according to the routine procedure of the department as described in details previously [8]. In brief: a small field-of-view gamma camera (GE starcam) was positioned in a left anterior oblique 30° view with a caudal tilt of 5°–10° adjusted for optimal separation of the ventricles. A bolus of 700–900 MBq of ^99m^Tc-labelled human serum albumin was injected. LVEF were calculated with GE software programs (entegra version 1.5; General Electric, Milwaukee, WI, USA). A subgroup of 73 patients had more than one examination (MUGA and BNP).

### BNP

Blood was drawn following 20 minutes of rest on the scanner bed prior to the administration of tracer and the BNP concentration was measured in plasma using an ADVIA Centaur two-side sandwich immunoassay technique based on chemiluminescence (Bayer, Leverkusen, Germany) [8], [19]–[21]. BNP cut-off value of 100 pg/mL is routinely used for diagnosing chronic heart failure in the general population [22] and has also been suggested for chemotherapy-related cardiotoxicity [7].

### Statistical analyzes

Differences in age, LVEF and BNP according to development of cardiac failure and all-cause mortality were analyzed using unpaired-sample t test. The plasma concentration of BNP were log-transformed before analyzes to ensure normal distribution. The chi-squared test was used for categorical data with dichotomized values for BNP and LVEF. Receiver operating characteristic (ROC) curves were drawn for plasma BNP concentration and LVEF to predict an adverse outcome (CHF or death, respectively) and area under the curves (AUC) were calculated. The prognostic value of BNP and LVEF for predicting either CHF or mortality was studied using Kaplan Meier method and significance was tested using the log rank test. Only (time to) the *first* hospitalization for CHF were included in analyzes. Univariate and multivariate analyses were carried out using Cox proportional hazards regression with age, BNP and LVEF included as covariates. BNP and LVEF were analyzed both as continuous and dichotomized values. Statistical analyses were performed with the use of IBM SPSS (version 20.0).

## Results

In total 333 patients (mean age 49, range 11–85 years) were included in the study. The mean follow up was 1,360 days (range 1–2,534 days). Tumor types were breast cancer (24%), various hematological malignancies (35%), uterine/ovarian cancer (10%). The remaining had a wide variety of miscellaneous types of tumors (30%).

During the follow up period 21 patients were admitted to hospital due to a diagnosis of congestive heart failure (CHF) and 239 patients died. Values of age, BNP and LVEF are listed for patients divided in groups according to CHF and all-cause mortality in table 1. BNP levels were significantly increased and LVEF were significantly lower in the group of patients that were subsequently admitted to hospital for CHF. No significant differences were observed (besides age) between survivors and patients that died during the follow up.

**Table 1:** BNP concentration and left ventricular ejection fraction (LVEF) in patients grouped according to the major end points; hospitalization for congestive heart failure (CHF) and death.

	BNP (pg/ml)	LVEF (%)	Age (years)
CHF (n = 21)	267,8 (107)	51,4 (3)	55 (4)
No CHF (n = 312)	41,3 (4)	61,1 (1)	49 (1)
*p value*	*0,000*	*0,000*	*0,139*
			
Dead (n = 194)	65,4 (13)	42,1 (8)	58 (1)
Survivors (n = 139)	61,6 (1)	59,1 (1)	41 (2)
*p value*	*0,158*	*0,743*	*0,000*

Results are given as mean ± standard deviation (SD).

**Figure 1:**
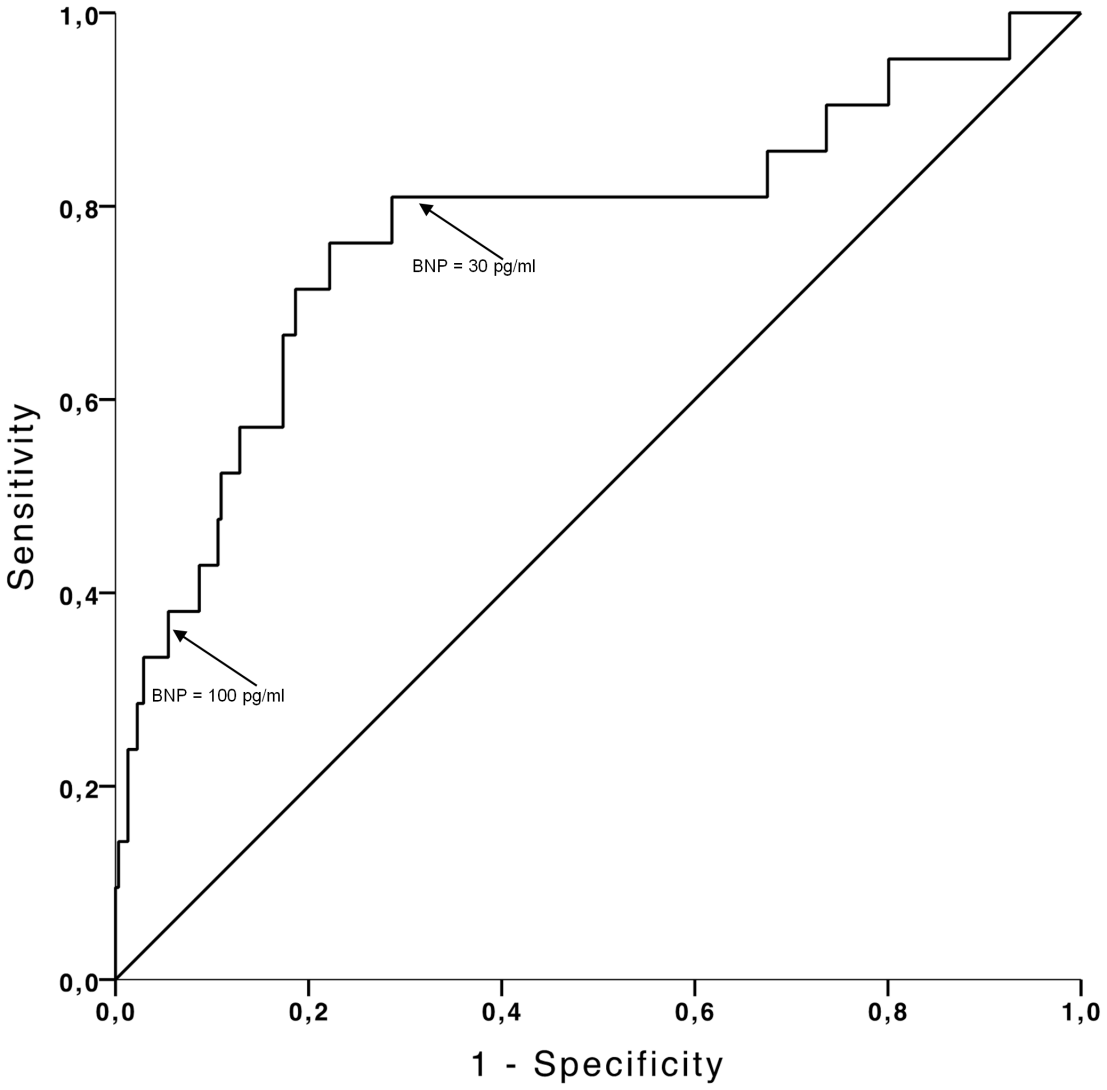
Received operating characteristic (ROC) curve for detection of CHF by BNP in patients treated with cardiotoxic chemotherapy. Area under the curve is 0.77 (95% CI 0.65–0.90, p<0.001). The arrows indicate selected cut-off concentrations of BNP. BNP concentration of 100 pg/ml correspond to a sensitivity 38% of and a specificity of 92% and BNP concentration of 30 pg/ml corresponds to a sensitivity 81% of and a specificity of 62%.

**Figure 2:**
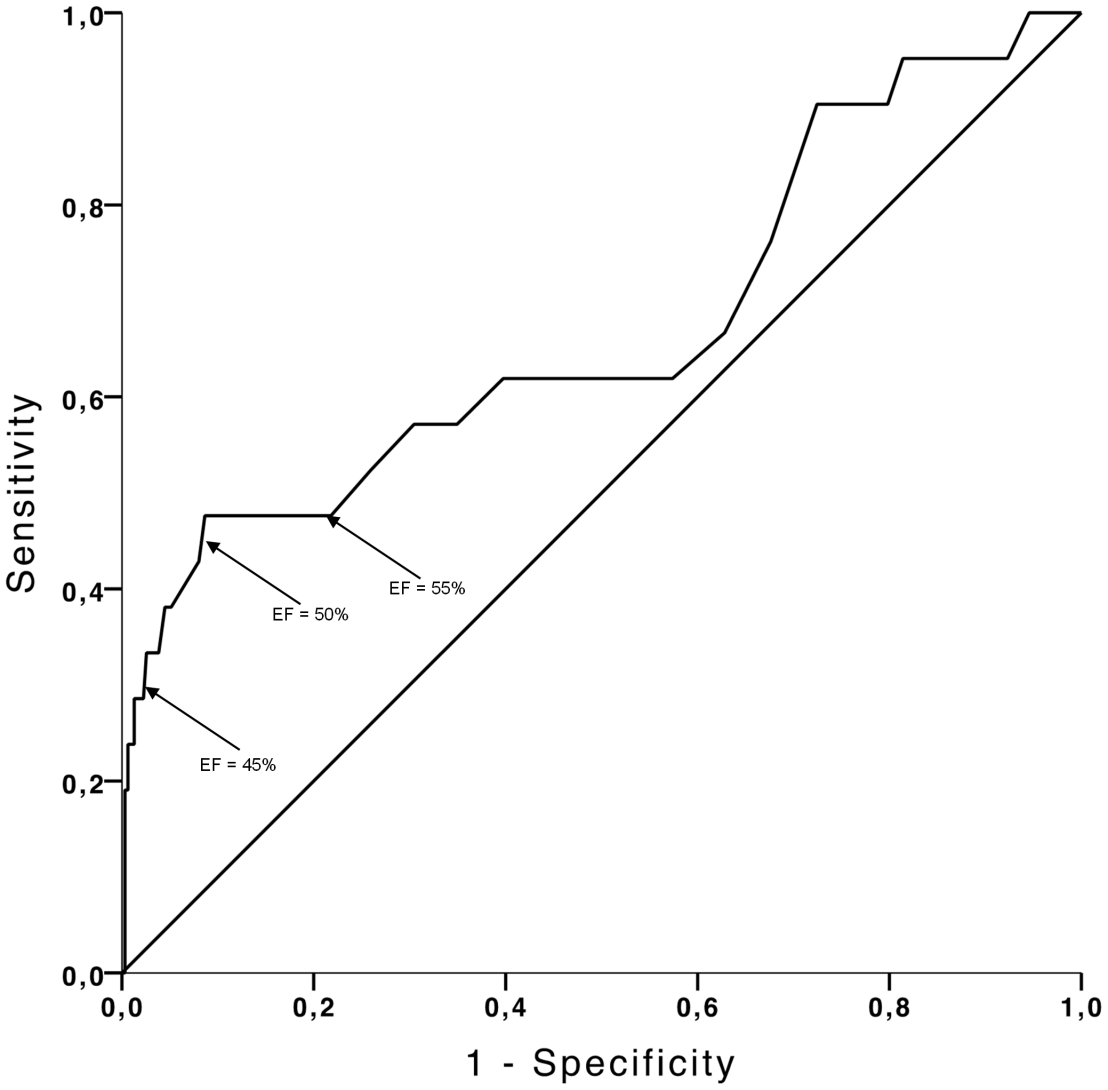
Received operating characteristic (ROC) curve for detection of CHF by LVEF in patients treated with cardiotoxic chemotherapy. Area under the curve is 0.67 (CI 0.53–0.81, p = 0.008). The arrows indicate selected cut-off concentrations for LVEF. A LVEF of 50% correspond to a sensitivity of 48% and a specificity of 91%. The diagnostic performance of LVEF at 45% and 55% showed a sensitivity of 38% and 45% and specificity of 97% and 78%, respectively.

Figure 1 shows the receiver operation characteristic curve for BNP for the diagnosis of CHF. The area under the ROC curve was 0.77 (95% CI 0.65–0.90, p<0.001). BNP at the routinely used cut-off value 100 pg/ml showed a sensitivity of 38% and a specificity of 92% for detecting CHF (table 2). Decreasing the BNP cut-off level to 30 pg/ml increased the sensitivity (81%) and decreased the specificity (62%) (table 2). When LVEF was used for detection of CHF (figure 2) the area under the ROC curve was 0.67 (CI 0.53–0.81, p = 0.008). A cut-off value of 50% of LVEF corresponding to the currently recommended cut-off value for detection of cardiotoxicity related to chemotherapy [2] resulted in a sensitivity of 48% and specificity of 91% (table 2). A cut-off value of LVEF at 45% resulted in a lower sensitivity (38%) and a high specificity (97%) in discriminating between patients with and without subsequent CHF (table 2), while increasing the cut-off value of LVEF to 55% resulted in a sensitivity of 48% and a lower specificity of 78% (CI: 0.27–0.69 and 0.77-0.80, respectively; p = 0.0130) (table 2). The ability of both BNP and LVEF to detect overall death assessed by ROC curve analysis revealed a low accuracy with AUC<0.6. BNP: AUC 0.58 (CI 0.52–0.64, p = 0.013) and LVEF: AUC 0.56 (CI 0.53–0.66, p = 0.002) (ROC curves according to overall death are not displayed).

**Table 2:** Prognostic performances of BNP and LVEF in predicting hospitalization for congestive heart failure during follow up.

CHF				
	BNP>100 pg/ml	BNP>30 pg/ml	LVEF<50%	LVEF<45%
Sensitivitet	**38** (20–59)	**81** (58–94)	**48** (27–68)	**29** (13–72)
Specificitet	**92** (91–93)	**62** (61–63)	**91** (90–93)	**98** (97–99)
PPV	**24** (13–38)	**13** (09–15)	**27** (16–39)	**46** (21–72)
NPV	**96** (94–97)	**98** (96–99)	**96** (95–98)	**95** (94–96)
*p value*	*0.000*	*0.000*	*0.000*	*0.000*

Values in ( ) are 95% confidence interval.

Table 2 and table 3 give the sensitivity, specificity, positive and negative predictive values of selected cut-off values of BNP and LVEF for the detection of clinical manifest congestive heart failure and overall mortality. Irrespectively of the chosen cut-off values the negative predictive value of both BNP and LVEF were high (>95%) in selecting patients developing congestive heart failure, while the sensitivity and positive predictive value was moderate. The diagnostic performance of BNP for detection of overall death at the cut-off level of 100 pg/ml showed a low sensitivity (13%) and a high specificity (94%) and lowering cut-off level to 30 pg/ml (a cut-off level also included in our initial analyzes of the baseline data [8]) resulted in higher sensitivity (81%) concurrently with a lower specificity (62%). Regardless of the cut-off values (45%, 50%, 55%) LVEF was non-significant for prediction of overall death by Fischers exact test.

**Table 3:** Prognostic performances of BNP and LVEF in predicting death during follow up.

Death				
	BNP>100 pg/ml	BNP>30 pg/ml	EF<50%	EF<45%
Sensitivitet	**13** (10–15)	**48** (43–52)	**11** (08–14)	**05** (03–06)
Specificitet	**94** (90–97)	**69** (63–75)	**89** (85–93)	**97** (94–99)
PPV	**76** (58–88)	**68** (61–75)	**59** (43–74)	**69** (39–90)
NPV	**44** (42–46)	**49** (44–53)	**42** (40–44)	**42** (41–43)
*p value*	*0.032*	*0.002*	*0.87*	*0.569*

Values in ( ) are 95% confidence interval.

**Figure 3:**
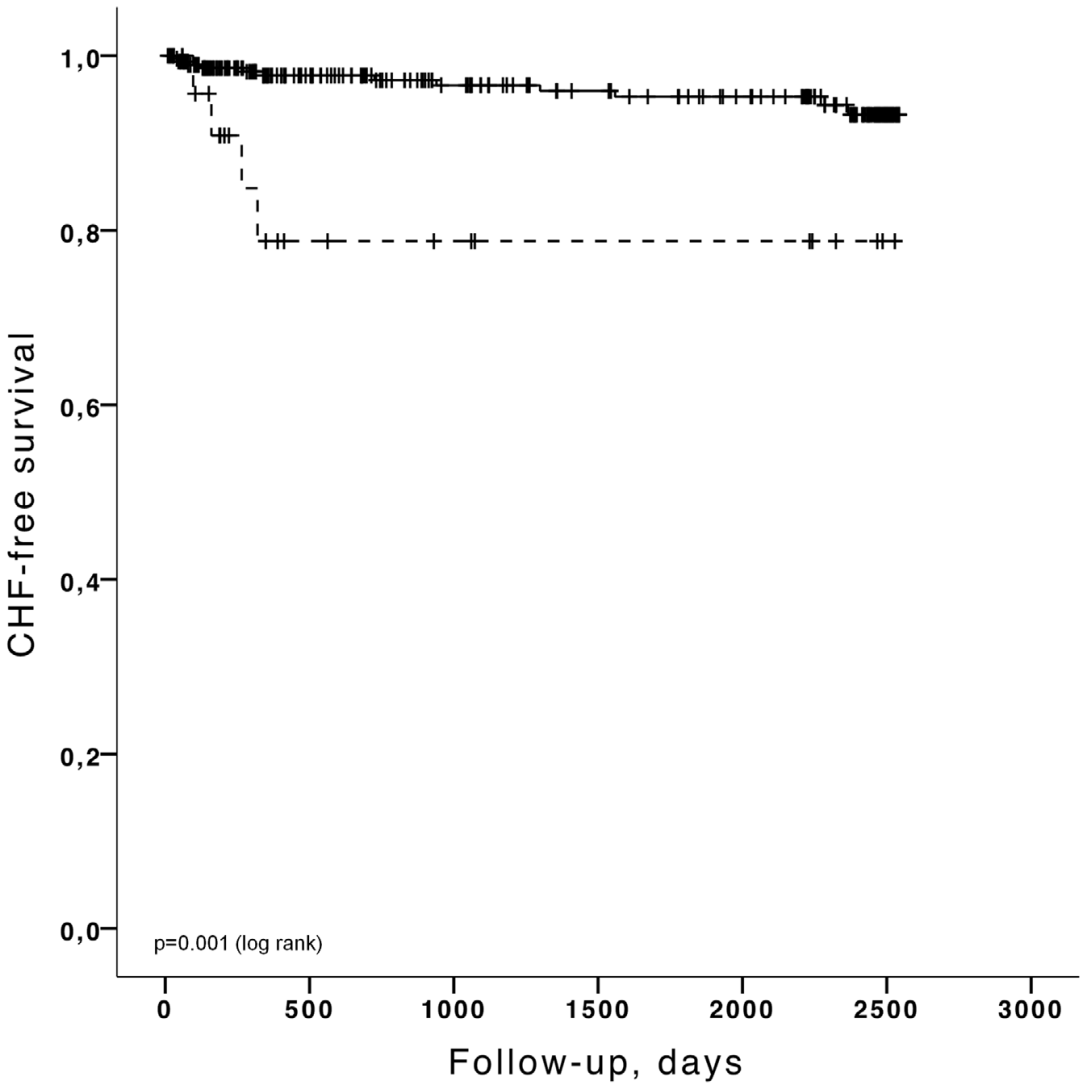
Kaplan-Meier analysis for the probability of CHF hospitalization-free survival according to BNP levels below (black, solid) or above the cut-off level of 100 pg/ml (black, dashed).

**Figure 4:**
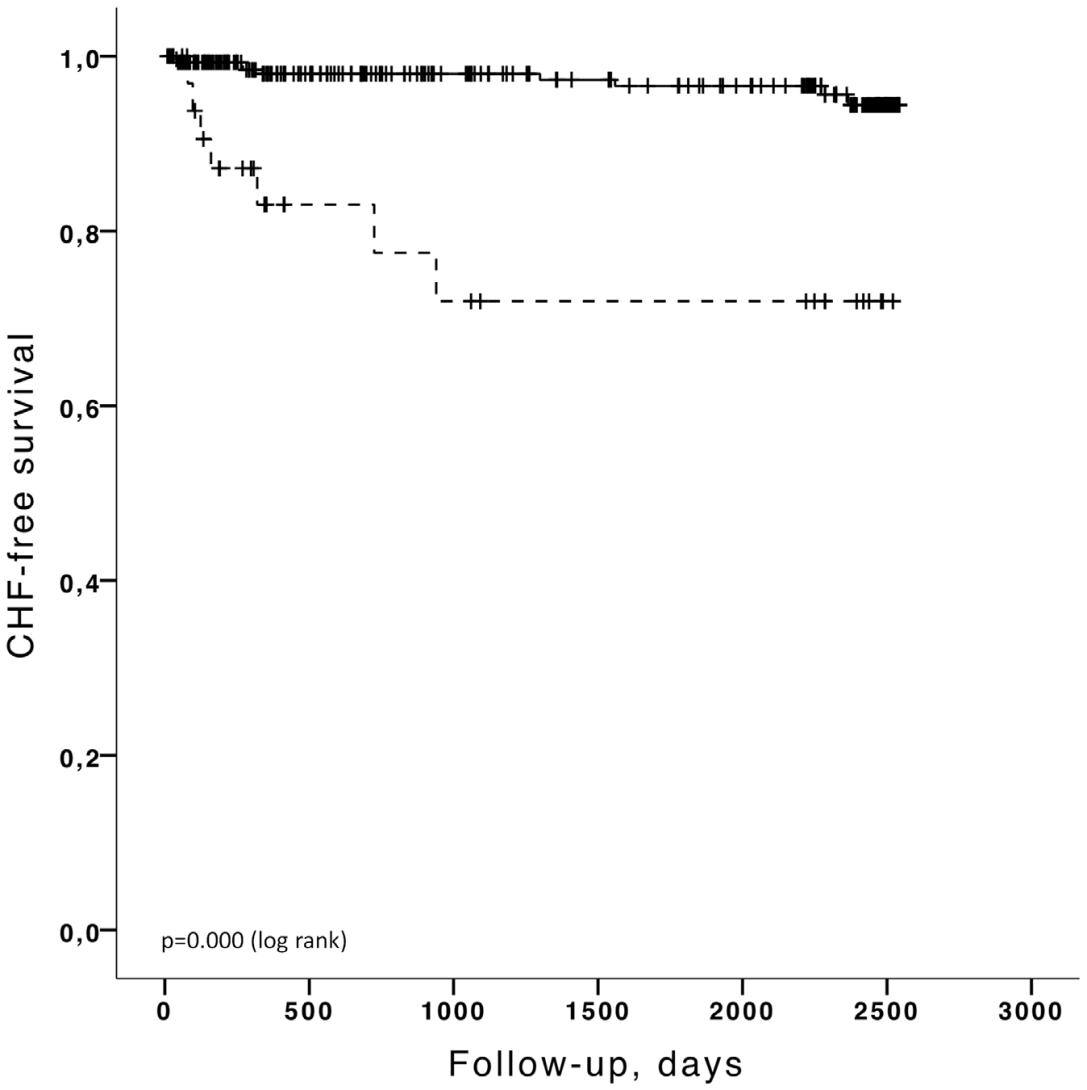
Kaplan-Meier analysis for the probability of CHF hospitalization-free survival according to LVEF above (black, solid) or below 50% (black, solid).

**Figure 5:**
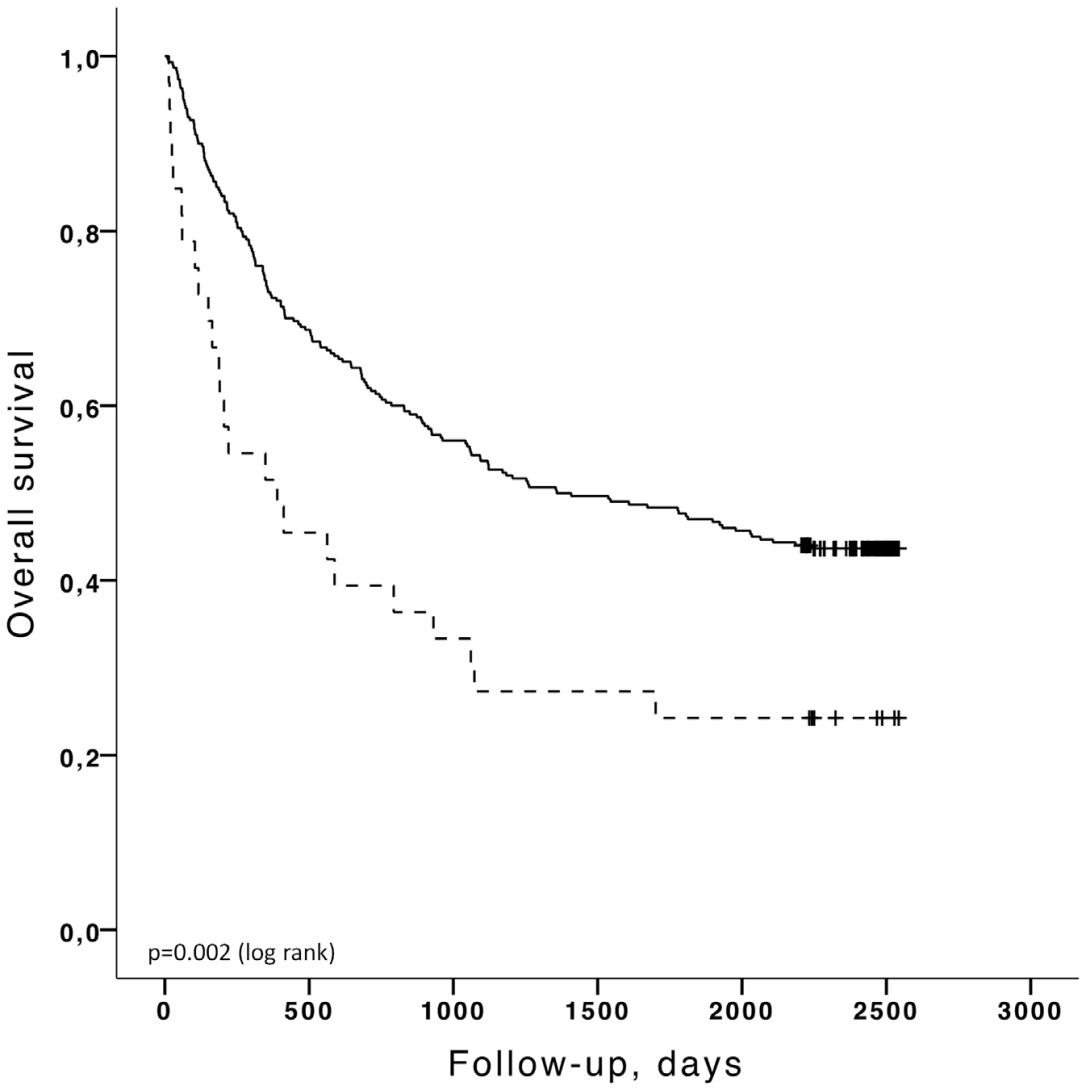
Kaplan-Meier analysis for the probability of over-all survival according to BNP levels below (black, solid) or above the cut-off level of 100 pg/ml (black, dashed).

**Figure 6:**
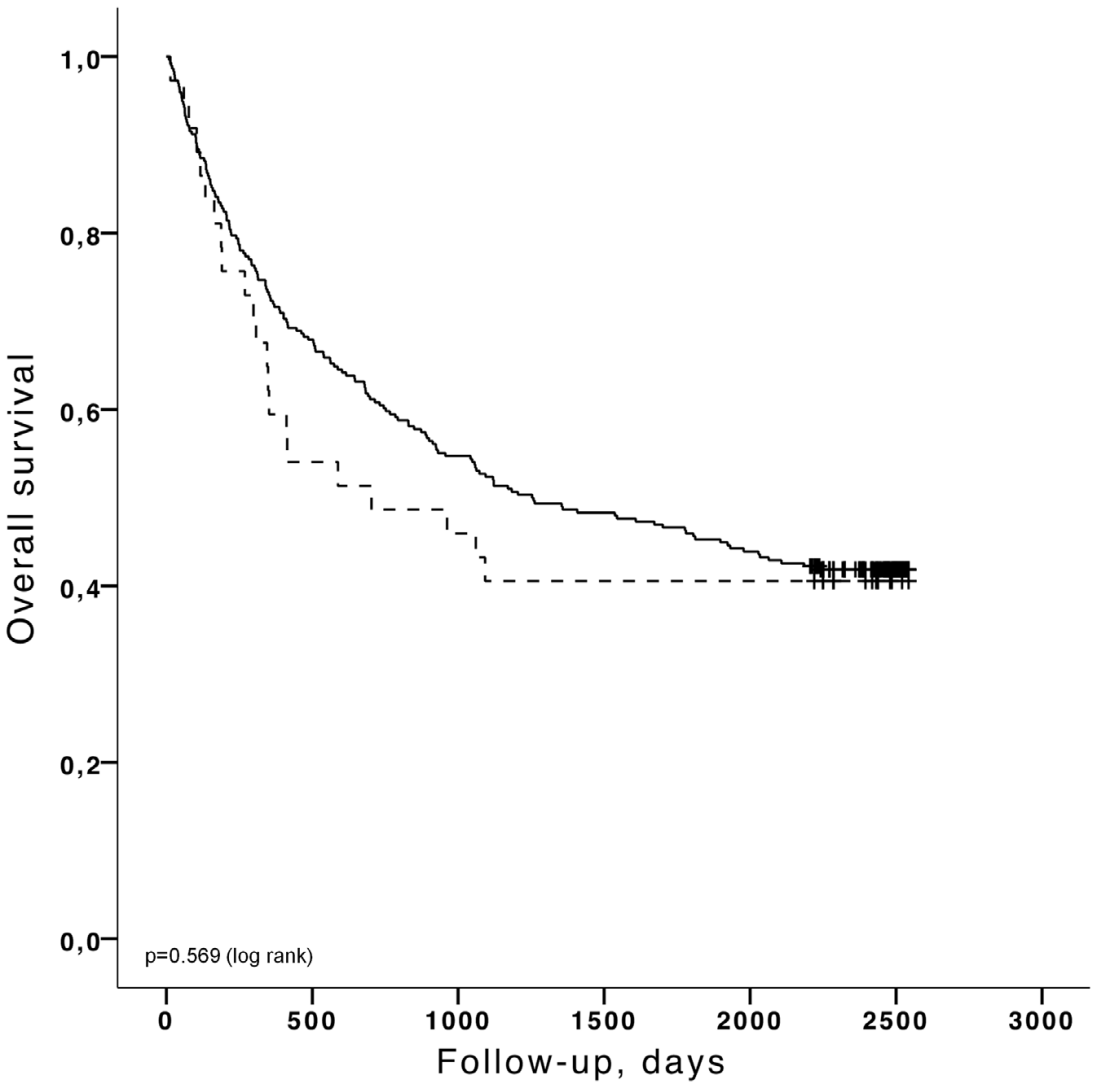
Kaplan-Meier analysis for the probability of over-all survival according to LVEF above (black, solid) or below 50% (black, dashed).

Kaplan-Meier analyses were carried out for the major end points (time to the *first* hospitalization for CHF and time to death) stratified by the selected cut-off values as described above. The Kaplan-Meier curves for cut-off values of BNP>100 pg/ml and LVEF<50% are shown in figures 3–6. Patients with a concentration of BNP>100 pg/ml had a higher risk of developing CHF (figure 3) (log rank p<0.001) and BNP >100 pg/ml was also predictive of overall death (figure 5) (log rank p = 0.003). LVEF<50% was significantly linked to CHF by Kaplan Meier analysis (figure 4) (log rank p<0.001) but LVEF<50% was not predictive of overall death (figure 6) (log rank p = 0.584). Patients with BNP values above 30 pg/ml had a higher risk of CHF (log rank p<0.001) and a higher mortality rate (log rank p<0.001). If the cut-off value for LVEF was selected to <45%, LVEF could predict CHF (log rank p = 0.000) but was still not able to differentiate survivors from non survivors (log rank p = 0.351). The unadjusted hazard ratios for the selected cut-off values for prediction of CHF and overall mortality based on univariate Cox regression analysis for age, BNP and LVEF are given in table 4 and table 5.

**Table 4:** Univariate Cox regression analysis for predicting hospitalization for congestive heart failure in relation to selected cut-off values of BNP concentration and LVEF.

CHF			
	HR	95% CI	*p value*
BNP	6.94	2.92–16.50	*0.000*
LVEF	0.000	0.00–0.032	*0.000*
BNP>100 pg/ml	5.51	1.76–17.22	*0.003*
BNP>30 pg/ml	6.02	1.94–18.74	*0.002*
LVEF<50%	7.95	2.95–21.41	*0.001*
LVEF<45%	11.11	2.98–41.44	*0.000*
Age	1.03	1.00–1.07	*0.053*

**Table 5:** Univariate Cox regression analysis for predicting overall death in relation to selected cut-off values of BNP concentration and LVEF.

Death			
	HR	95% CI	*p value*
BNP	1.68	1.26–2.34	*0.000*
LVEF	1.76	0.32–9.68	*0.519*
BNP>100 pg/ml	1.94	1.72–2.96	*0.002*
BNP>30 pg/ml	1.70	1.28–2.25	*0.000*
LVEF<50%	1.14	0.73–1.78	*0.569*
LVEF<45%	1.08	0.53–2.25	*0.823*
Age	1.04	1.03–1.05	*0.000*

In a multivariate Cox proportional regression analysis, with age, LVEF and BNP as continuous and dichotomized variables, both LVEF<50% and BNP were statistically significant independent predictors of CHF (LVEF:<50% HR 4.89; CI 1.49–13.53, p = 0.008 and BNP: HR 4.16, CI 1.63–10.42, p = 0.002). When only dichotomized values of BNP and LVEF were entered in the model BNP>30 pg/ml and LVEF<50% remained statistically significant for detection of CHF (BNP>30 pg/ml: HR 4.87, CI 1.55–15.36, p = 0.007 and LVEF<50%: HR 6.13, CI 2.25–16.68, p = 0.000). In a multivariate analysis of BNP and LVEF as dichotomized values according to overall death, age was the only significant independent predictor of overall death (HR 1.04, CI 1.03–1.05, p = 0.000). In addition BNP>100 pg/ml showed a borderline significant value of 0.077 as an independent predictor of overall death (HR 1.47, CI0.96–2.24).

Of the 73 patients with more than one examination no changes in BNP concentration (46.6±9.0 vs. 43.4±7.3 pg/ml, p = 0.544) and no changes in LVEF (0.58±0.01 to 0.58±0.01, p = 0.37) were found from the first to the last examination. In this subgroup, 5 patients were subsequently hospitalized with CHF. Only one of these 5 patients had a significant increase in BNP from below to above the cut-off of 100 pg/ml and in none of the patients, LVEF fell >10% point from the first to the last examination. For the whole group of multi-examined 73 patients, neither an increase in BNP (n = 36) nor a decrease in LVEF of more than 0.10%-point from the first to the last examination or a final LVEF determination of less than 0.50 (*n* = 10), predicted CHF (BNP: HR 0.63, CI 0.11–3.78, p = 0.61 and LVEF: HR 0.04 CI 0.0–2561, p = 0.57).

## Discussion

The present study prospectively investigated BNP concentration and LVEF as predictors of two distinct clinical endpoints (hospitalization for manifest congestive heart failure and overall death) in 333 anthracycline-treated cancer patients. The most important finding was that both BNP and LVEF independently predicted congestive heart failure. However, only BNP and not LVEF was associated with overall mortality. Although the level of BNP can be influenced by e.g. adipositas, pulmonary and renal disease and drugs such as angiontensin converting enzyme inhibitors, BNP can predict over-all mortality in a wide range of other patient categories such as hypertension, stroke and diabetes [23]

In the literature the reported incidence of cardiovascular toxicity from anticancer treatment is highly variable and depends on the type of drug being used. The patients in the study cohort were treated with anthracycline-containing chemotherapy regimens. Anthracyclines are widely used and have well-known cardiotoxic effects with a reported incidence of 0.9–26% [4]. The cumulated incidence of clinical manifest congestive heart failure in the current study was 6.3% for the entire ∼4 year follow up. The relatively high mortality rate (58% died) during the follow-up period most likely reflects aggressive/disseminated malignant disease in the study population.

In this cohort of cancer patients, 21 of the 333 patients were admitted to hospital for congestive heart failure during the follow-up period. If the current recommendations for surveillance of cardiotoxicity in relation to chemotherapy are followed where cardiotoxicity is defined by LVEF<50% (determined by a single examination), only 48% (10 patients) were correctly identified of developing CHF and accordingly 52% of the patients (11 patients) with subsequent cardiac failure were overlooked. Increasing the threshold of LVEF to <55% did not change the number of heart failure patients that would have been identified (10 patients with cardiac failure had LVEF<55%). In comparison a BNP>30 pg/ml could identify 79% (17 patients) of these patients overlooking 21% (4 patients). This emphasizes that the current method based on LVEF is suboptimal in detection of subtle alterations in left ventricular function and that even small increases in BNP (below the recommended routinely used cut-off value) might be applicable in identifying patients at risk of developing cardiac dysfunction. At the routinely used cut-off value of BNP (>100 pg/ml) a substantial part of the patients with subsequent heart failure were not identified (13 patients corresponding to 62%) suggesting that the cut-off value should be decreased if measurements of BNP is applied in the monitoring of cardiotoxicity. However, a major challenge is that 135 patients had BNP levels above 30 pg/ml, resulting in a substantial number of “false positives”. This probably reflects a moderate transient increase in BNP during treatment due to acute/subacute cardiotoxicity that only in some patients are irreversible [7]. It was speculated if BNP could serve as a “gatekeeper” implicating that only patients with BNP>30 pg/ml were referred to the more expensive and laborious estimation of LVEF by MUGA. If the 135 patients with BNP >30 pg/ml were subjected to MUGA and LVEF<50% again was considered reference value, then only 8 of the 21 heart failure patients would have been identified. Another possibility could be to use BNP as an additive measure together with LVEF-measurement in the surveillance of cardiotoxic cancer treatment. If these two values (patients having either LVEF<50% *or* BNP>30 pg/ml) were combined 18 of the 21 patients with CHF were identified suggesting that BNP possess meaningful and additive information to the current surveillance based on MUGA. However, by combining BNP and MUGA only 1 more patient was identified compared to BNP>30 pg/ml alone and 150 patients had either BNP>30 pg/ml or EF<50, increasing the number of “false positives” compared to BNP>30 pg/ml as the only measure.

It was also speculated if serial measurements of BNP could have improved the diagnostic performance of chemotherapy-induced CHF, since it is well known that repeated measurements in patients with chronic heart failure provide incrementally unique information [22]. Unfortunately, this was only done in 73 of the patients, of whom 5 patients developed congestive heart failure, making the material too small for meaningful statistical analyses.

Several published studies have investigated the role of BNP in diagnosing and risk stratification of chemotherapy-associated cardiotoxicity, however most studies have simply compared BNP levels with LVEF measured by MUGA or echocardiography at baseline or during/after treatment with antracyclines as we did also in our previous publication [8]. Results have shown either no association [10]–[12] or a correlation between impaired ejection fraction and increased levels of BNP [9], [13], [24].

There are multiple reasons for these conflicting results including inadequate sample size (most studies include <100 patients), heterogeneity of the studied population with regards to cancer diagnosis, age (adults vs. children), chemotherapeutic agent and treatment regime/cumulative dose. Furthermore, the timing of blood samples drawn for BNP measurements, the laboratory methods and cut off values contributes to the inconsistency of the results. In this study BNP was measured at same the session where MUGA was performed which typically is when half of the cumulative dose of anthracyclines has been given.

Only a few published studies have investigating the use of BNP as predictor of a true clinical end-point such as manifest congestive heart failure. In a study by Pichon et al [7], BNP was correlated to clinical heart failure (as classified by NYHA) in 67 breast cancer patients followed for 62 days (1-984 days) after chemotherapy (anthracyclines with/without trastuzumab) and cut-off level 100 pg/ml was suggested for prediction of CHF. Lee et al [16] studied 86 patients with hematologic malignancies for 2 years and found a correlation between increased BNP concentration and CHF (as diagnosed by Framingham criteria) following antracycline treatment and also Feola et al [6] found that BNP predicted cardiac events (based on reported symptoms or decrease in LVEF>10% at two year follow-up) in 53 breast cancer patients. Other studies focusing on clinical end points have been small including <20 patients [13], [25].

Several studies have used NT-ProBNP which despite a longer half-life are considered comparable to BNP [22]. Due to differences in conclusion, study design and importantly the majority of the studies are based on <50 patients it is difficult to draw definite conclusions [26]–[31].

In addition, a limited number of studies have investigated the possibility of using BNP and other biomarkers in the early detection of anthracycline-induced cardiotoxicity in children/long-term survivors of childhood cancer with the majority of studies supporting BNP as a useful biomarker of myocardial dysfunction [32]–[36].

Importantly, the current results presented here, based on the hitherto largest cohorts of patients (333 patients) investigating BNP as a predictor of clinical manifest congestive heart failure, clearly show that BNP has an important predictive value comparable or even superior to the currently recommended more time-consuming, radiation exposure involving and expensive MUGA.

There are some important limitations to the study; the incidence of congestive heart failure in the present study might be a conservative estimate since only clinical manifest congestive heart failure treated in hospital and outpatient clinics are recorded. It is possible that we have overlooked patients with subclinical cardiac impairment or patients with only vague clinical symptoms, which would not be categorized as CHF (DI1427 or DI50-509) in the Danish national patient registry.

Furthermore, we have no detailed information on the chemotherapeutic regime, including the cumulative dose of anthracyclines and/or addition of thoracic radiotherapy. This is notable since the cumulative dose of anthracyclines and the addition of other cardiotoxic chemotherapeutics including trastuzumab and also thoracic radiotherapy are established risc factors for developing chemotherapy-related CHF [1]. No baseline medical history with respect to cardiovascular risk factors or concurrent administration of cardiovascular drugs was obtained. Recently, several studies have shown that adding cardioprotectans such as ACE-1 or Beta-blockers can prevent LVEF reductions in patients undergoing intensive chemotherapy [37]. Lastly, other specific cardiac biomarkers that are reported to detect subclinical chemotherapy-associated cardiac damage (e.g. the troponins, with a high sensitivity for detecting small amounts of myocardial necrosis) were not measured in the current study.

## Conclusion

In conclusion, this is the largest patient data set (333 patients) reported till date investigating both plasma BNP concentration and LVEF as predictors of chemotherapy-related cardiotoxicity using distinct clinical end points: hospitalization with a diagnosis of congestive heart failure and overall death. This prospective study shows that for cancer patients treated with cardiotoxic chemotherapy both BNP and LVEF significantly predicted congestive heart failure. Only BNP and not LVEF had diagnostic implications in predicting overall mortality supporting BNP as a clinical relevant factor for monitoring chemotherapy-related cardiac toxicity and death.

A future prospective clinical trial should focus on standardization of the use of BNP concentration for diagnosing patients with irreversible cardiac damage, including determining optimal cut-off level and timing of BNP requiring several samples. In addition, a future focus should be on therapeutic-decision making (e.g. dose adjustment/addition of cardioprotective medical therapy) based on BNP concentration compared to the current approach with assessment of LVEF ideally in a randomized study.
